# Adventurous Play as a Mechanism for Reducing Risk for Childhood Anxiety: A Conceptual Model

**DOI:** 10.1007/s10567-020-00338-w

**Published:** 2021-01-19

**Authors:** Helen F. Dodd, Kathryn J. Lester

**Affiliations:** 1 School of Psychology and Clinical Language Sciences, University of Reading, Whiteknights, Reading, RG6 6AL UK; 2 School of Psychology, University of Sussex, Falmer, Brighton, BN1 9QH UK

**Keywords:** Play, Risky play, Child anxiety, Fear, Adventurous play, Risk

## Abstract

In this conceptual article, we draw upon the literature regarding cognitive and behavioural factors that underpin childhood anxiety to outline how a range of these risk markers might be targeted through adventurous play. When children play in an adventurous way, climbing trees, riding their bikes fast downhill and jumping from rocks, they experience feelings of fear and excitement, thrill and adrenaline. We propose that the positive, thrilling and playful emotions associated with this type of child-led play facilitate exposure to fear-provoking situations and, in doing so, provide opportunities for children to learn about physiological arousal, uncertainty and coping. We hypothesise that these learning opportunities will, over time, reduce children’s risk for elevated anxiety by increasing children’s expectations and ability to cope with anxiety, decreasing intolerance of uncertainty and preventing catastrophic misinterpretations of physiological arousal. If our conceptual model is correct, then ensuring that children have the physical and psychological space required to play in an adventurous way may help to decrease their risk for elevated or clinical anxiety.

It is well established that trajectories to anxiety disorders begin early in life (Caspi et al. 1988). Half of all anxiety disorders begin before the age of 11 and elevated anxiety symptoms early in life predict subsequent anxiety disorders (Hudson and Dodd 2012; Kessler et al. 2005). Developmental models of anxiety psychopathology (e.g. Manassis and Bradley 1994; Vasey and Dadds 2001) have stimulated extensive research directed at understanding and delineating pathways of risk and resilience for anxiety. Childhood anxiety is associated with a range of cognitive and behavioural factors including child temperament, specifically behavioural inhibition (Chronis-Tuscano et al. 2009; Hudson and Dodd 2012; Lahat et al. 2011), avoidance (Craske 2003), over-involved parenting (Hudson et al. 2011; McLeod et al. 2007), intolerance of uncertainty (Osmanağaoğlu et al. 2018), a bias to interpret ambiguity in a negative way and a tendency to anticipate that they will not be able to cope in ambiguous situations (Dodd et al. 2015; Field and Lester 2010; Stuijfzand et al. 2018). To decrease children’s risk for developing high levels of anxiety or an anxiety disorder, prevention and early intervention programmes have been designed to target these factors (e.g. Chronis-Tuscano et al. 2015; Kennedy et al. 2009). In this conceptual paper, we outline a model (see Fig. 1) which hypothesises that adventurous play during early and middle childhood (ages 3 to 11 years) can target these same cognitive and behavioural factors and, as a result, decrease children’s risk for anxiety.

**Fig. 1:**
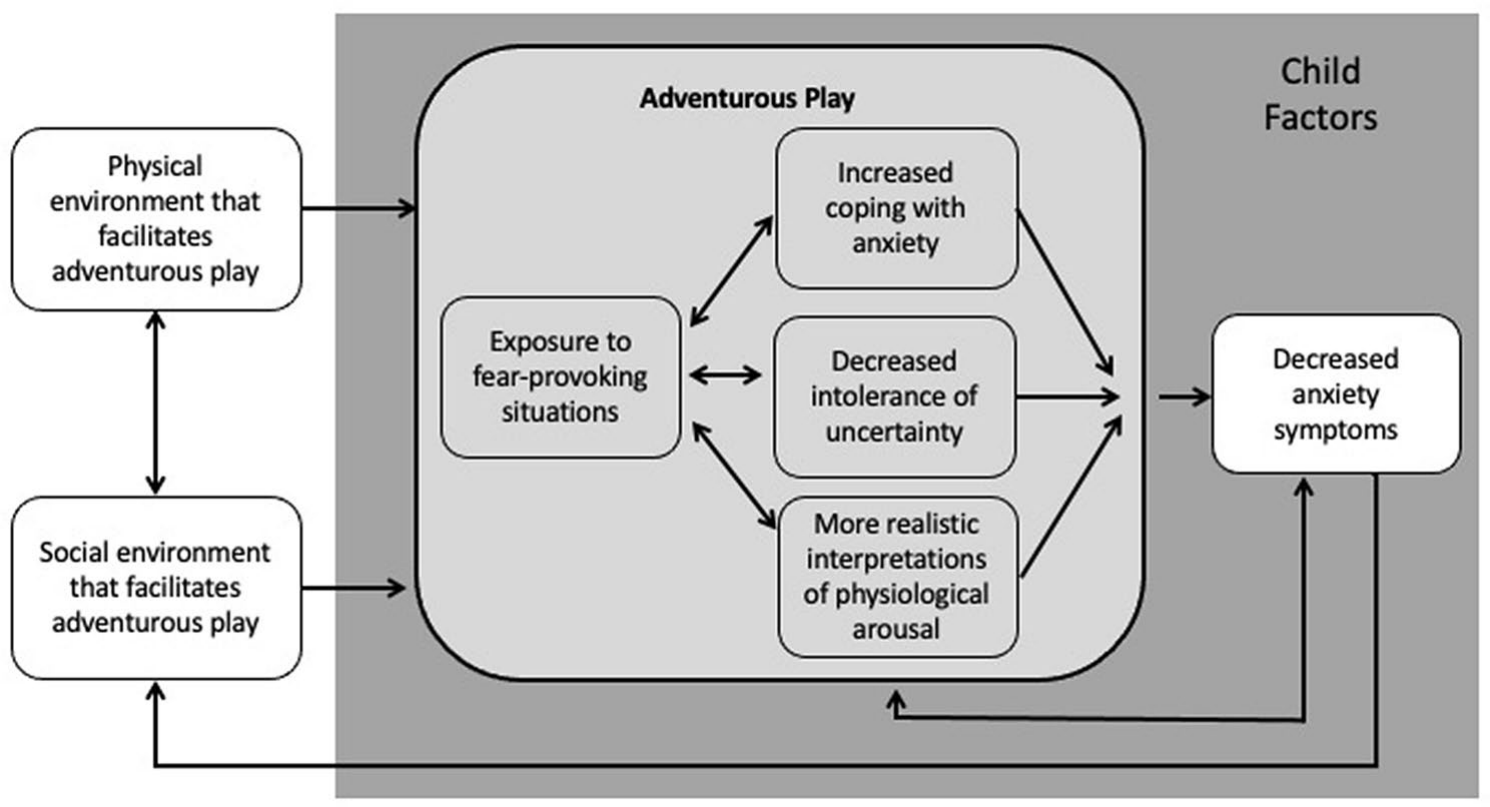
A novel psychological model capturing how environmental and social factors affect children’s adventurous play and how, in turn, adventurous play might affect anxiety risk and anxiety symptoms via cognitive-behavioural factors proposed to underpin anxiety. This occurs within the context of the individual child. This is not intended to be exhaustive and we recognise that other environmental and social factors such as attachment, poverty and stress affect children’s play and that other factors might mediate any adventurous play-anxiety relationship

Adventurous play is defined as child-led play where children experience subjective feelings of excitement, thrill and fear; often this occurs in the context of age-appropriate risk-taking. We argue that this type of play provides opportunities for children to learn about uncertainty, fear, arousal and coping and describes the mechanisms through which we hypothesise that adventurous play might affect children’s risk for elevated and clinical anxiety. We focus on early to middle childhood because this is a crucial period for the initial development of anxiety-related problems in children, and the age group for whom prevention and early intervention for anxiety are typically designed. Furthermore, much of the research into risk factors and correlates of child anxiety focuses on this age range, providing an evidence base for our model. Our overarching hypothesis is that, if children are given ample opportunity, across childhood, to explore age-appropriate, healthy risk-taking through child-led adventurous play, they may learn skills that help them successfully navigate subsequent anxiety and fear-provoking situations; the focus is not on treatment for clinical child anxiety disorders.

Before describing the relevant literature and conceptual model, there are two caveats. The first is that the overall effect size of adventurous play on children’s risk for anxiety is likely to be small. Anxiety is underpinned by a broad range of risk factors and complex causal pathways, where multiple risk factors interact with one another to affect risk for anxiety. We propose that adventurous play may be one way of decreasing children’s long-term risk by providing important opportunities to learn about coping, uncertainty and physiological arousal, not that it provides a broad solution to child anxiety. Children will also learn via a wide range of other experiences such as modelling (Askew and Field 2008) and information transfer (Muris and Field 2010). In the context of the Vasey and Dadds (2001) developmental psychopathological model of child anxiety, we view adventurous play as a protective influence which interacts with other protective influences and predisposing factors to contribute to a child’s cumulative risk.

A second caveat is that we focus on child anxiety and specific cognitive and behavioural factors for which there is a plausible link with adventurous play. In our model, we have considered anxiety as a broad concept, but it is possible that adventurous play may be more beneficial in preventing specific subtypes of anxiety than others. For example, adventurous play may have less relevance for social anxiety than for generalised anxiety or phobias. It is also possible that this type of play is beneficial for other emotional and behavioural problems such as externalising problems. This is beyond the scope of the present paper but may be of interest in future research.

## Adventurous Play

Play is prolific in childhood. Children play at home, at the park, with friends, at school, even when they’re eating. There are few constructs as intimately entwined with childhood and as ubiquitous as play. Despite this, play as a broad topic is relatively neglected in psychological research relative to other aspects of child development. When scientific research does focus on play in children, it is dominated by work on pretend play (Lillard et al. 2013; Pellegrini et al. 2007). In contrast, psychological study of locomotor (or physical) play in children is rare, to such an extent that it has been claimed that ‘psychologists have ignored one of the most common forms of play’ (Pellegrini 2009, p. 137). This is surprising given that playgrounds are a staple of western neighbourhoods and it is estimated that children spend around 20% of their time engaged in locomotor play, which includes climbing, swinging, chasing, balancing, jumping and other playful physical activity (McGrew 1972; Smith and Connolly 1980).

In contrast to human children, locomotor play has been extensively studied in animals (e.g. Bekoff and Byers 1981; Fagen 1981; Povinelli and Cant 1995). Almost all mammals have been observed to spend some of their time engaged in locomotor play (Pellis and Pellis 2009). An important component of animal locomotor play appears to be to experience thrill through exposure to moderately fear-provoking situations. For example, primates leap and swing in trees and chase one another, playing in such a way that they appear to intentionally switch between being in and out of control, thus exposing themselves to moderate levels of fear and arousal (Spinka et al. 2001).

It has been proposed that, in juvenile animals, this type of play provides exposure to moderately fear-provoking stimuli and facilitates learning about how to cope emotionally with the unexpected (e.g. Pellis and Pellis 2009; Spinka et al. 2001). Specifically, Spinka et al. (2001) hypothesise that a major function of play is to provide a context where animals ‘lose full control over their locomotion, position or sensory/spatial input and need to regain these faculties quickly’. It is argued that when playing in this way animals learn not only ‘increased versatility of movements’ but also how to ‘avoid emotional overreaction during unexpected stressful situations…These may include locomotor shocks…and psychological shocks such as suddenly being faced with frightening or dangerous stimuli, unexpectedly meeting a stranger or experiencing a sudden reversal in dominance’ (p. 143). Similarly, Pellis and Pellis (2009) present evidence that indicates that play experience in rats may ‘fine-tune’ coping skills needed for managing emotional responses to social and non-social situations. To date, these ideas have not been explored empirically in humans, but it seems plausible that adventurous play serves a similar purpose in human children.

Human children’s play mirrors this type of play seen in animals; children also deliberately expose themselves to moderate levels of fear, for example, through play at heights or high speed (Sandseter 2009). Naturally, there are individual differences between children in what constitutes a risk and in desire for risk (see Morringiello and Ladenby-Lessard 2007 for a review) but a desire for some degree of thrilling play experience appears to be common across all children (Sandseter and Kennair 2011). This individual difference in desire for risk-taking aligns with the construct of sensation seeking and is likely to be an important child factor influencing children’s engagement in adventurous play.

Following theoretical ideas about the purpose of this adventurous or risky play in animals, it has been proposed that this type of play may serve a similar purpose in children (e.g. Gray 2011, 2013; Sandseter and Kennair 2011). For example, Gray (2013) argues that when children are free to play, they ‘deliberately put themselves into fear-inducing, vulnerable positions in their play…testing their own fear as well as their physical prowess’ (p. 172–173). This leads to thrilling feelings that come from simultaneous fear and joy. He argues that, through this type of play, children learn how to solve their own problems, control their impulses and modulate their emotions. Sandseter and Kennair (2011) provide a detailed theoretical account of how risky play, which is synonymous with adventurous play, may serve to expose children to stimuli that they previously feared. Sandseter and Kennair propose that through risky play, children naturally perform exposures, which function to reduce developmentally normal fears. Specifically, they argue that the positive, thrilling emotion associated with this type of play facilitates and motivates exposure to feared stimuli. This process of exposure mirrors the mechanisms that underpin successful exposure therapy for clinically diagnosed phobias (Hoffman 2008; Whiteside et al. 2020).

On the basis of Sandseter’s (2007) six categories of risky play (great heights, high speed, dangerous tools, play near dangerous elements, rough-and-tumble, play where children can disappear/get-lost), Sandseter and Kennair (2011) propose that specific categories of risky play have specific anti-phobic functions. For example, the category ‘Play with great heights’ is proposed to provide a desensitising experience and skill development that results in reduced fear of heights. Consistent with this hypothesis, and in the opposite direction to what would be predicted by conditioning theory, a prospective study showed that children who injured themselves falling from heights during middle childhood were subsequently less likely to report a fear of heights at age 18 (Poulton et al. 1998). Similarly, play where children can ‘disappear’ or get ‘lost’ is hypothesised to support exposure to separation. Again, broadly consistent with this idea, children who experienced more planned separation experiences from a caregiver between age 3 and 5 years (albeit not specific to play situations) had fewer separation anxiety symptoms later in childhood (Poulton et al. 2001). In very young children, play involving vicarious risk, where children observe others taking risks, is hypothesised to have the same arousing effect as ‘real’ risky play experiences and may act as a precursor to risky play, and with a potentially similar desensitising experience to feared stimuli (Apter 1992).

In this paper, we propose a conceptual model (see Fig. 1) that builds on Sandseter and Kennair’s (2011) ideas. Whereas Sandseter and Kennair focus solely on phobias and exposure, in this article we draw on the anxiety literature and propose that adventurous play provides a range of learning experiences that can target cognitive and behavioural mechanisms associated with anxiety in children. As a consequence, we hypothesise that this type of play may help to prevent elevated anxiety more broadly defined.

## Injury Prevention in the Context of Adventurous Play

Discussion of risk-taking in play often raises questions about injuries. In many countries, unintentional injuries are a leading cause of death in children (Peden et al. 2008) and there is understandable concern about protecting children from unnecessary risks. In the UK between 1980 and 2010, 31% of child deaths among 1–4-year-olds and 20% of child deaths among 5–14-year-olds were from unintentional injuries (Royal College of Paediatrics and Child Health 2013). Data on hospital attendance and admissions show that approximately 2 million UK children under the age of 15 attend Accident and Emergency, and 108,000 children require hospital admission each year due to unintentional injury (Child Accident Prevention Trust 2013); the estimated cost of treating children’s accidents amounts to more than £275 million a year (Royal Society for the Prevention of Accidents 2020).

While these are sobering statistics, the category of unintentional injury is broad and includes road traffic injuries as well as poising and fire-related injuries. The majority of children’s accidents happen in the home (Shanon et al. 1992). For young children, falls are the leading cause of injury-related hospital admissions (CDC 2019), with these most commonly happening from furniture and more serious accidents involving falls from windows, balconies and stairs (Consumer Safety Unit 2002). In contrast, research indicates that when children are playing, they display strategies for preventing serious injury (Christensen and Mikkelsen 2008) and recognise that major injuries communicate carelessness and clumsiness (Green 1997). Medically treated injuries during children’s play are very rare; for children aged 6–12 years, there are between 0.15 and 0.17 injuries per 1000 hours of physically active leisure time. This contrasts with 0.2–0.61 injuries per 1000 hours of organised sport. Thus, while we recognise that child injuries can be serious and associated with long-term costs, for children growing up in western society, the risk of being injured during play is very low (Nauta et al. 2015). In this context, a risk benefit approach is required which takes into account the benefits of challenging play experiences as well as the risks, ensuring children are not exposed to unnecessary danger but that they are given space to play in a way that is adventurous, stimulating and creative (Ball et al. 2012; Gill 2018).

A caveat here is that certain groups of children are over-represented in injury statistics, and some children are continually exposed to riskier, more dangerous environments than others (Giles et al. 2019). For example, children living in poverty and marginalised communities are more likely to be affected by unintentional injuries; compared to children whose parents are employed in higher professional occupations, children of parents who are long-term unemployed or who have never worked are 13 times more likely to die from an unintentional injury (Towner et al. 2005). Similarly, children living in impoverished and marginalised communities are more likely to experience unintentional injury both in and outside of the home (Balan and Lingam 2012). They also often have fewer opportunities for play due to unequal access to safe parks and playgrounds and age-appropriate extracurricular activities (Barnes 2012; Milteer et al. 2012). While we hypothesise that there will be benefits of age-appropriate, healthy risk-taking through child-led adventurous play, as Giles et al. (2019) highlight, this is unlikely to be relevant for children who are exposed to chronic levels of risk. We therefore acknowledge that our hypothesis may be most relevant for children who are growing up in relative safety.

## Cognitive–Behavioural Factors Hypothesised to be Affected by Adventurous Play

Research on childhood anxiety disorders has established a range of cognitive–behavioural factors that underpin dysfunctional anxiety and/or are implicated in the aetiology of anxiety. Anxiety is multifaceted and causal pathways are complex. We propose that adventurous play could target some of the specific cognitive–behavioural factors associated with child anxiety, as shown in Fig. 1, and in doing so, it could decrease children’s risk for anxiety in the long term. Each of these factors will now be discussed in turn.

### The Importance of Exposure Rather than Avoidance

Avoidance is critical to theories of anxiety and is hypothesised to play a role in both the development of anxiety and the maintenance of anxiety over time (Craske 2003; Hudson and Rapee 2004; Manassis and Bradley 1994). Behavioural avoidance, characterised by moving away from a threat, serves an adaptive function when threat level is high, reducing the potential for harm. However, when an individual perceives a stimulus or situation to be more dangerous than it objectively is, avoidant responses prevent exposure to the feared situation and the learning opportunities that then follow. When a feared situation is experienced, rather than avoided, this offers an opportunity for threat and coping appraisals to be challenged and adjusted. Exposure supports fear extinction (Craske 2003) and the gathering of information which can challenge misappraisals and disconfirm predictions of fear and negative outcomes (Rudaz et al. 2017).

Support for the importance of exposure to feared situations and stimuli comes from research with children who are Behaviourally Inhibited (BI). Children who are BI respond to novel and unfamiliar situations with avoidance and wariness (Kagan et al. 1984). Due to the tendency to respond with avoidance, children who are BI have reduced opportunity for exposure to feared stimuli and the learning opportunities that this brings. Hudson and Rapee (2004) propose that this tendency, combined with parental support of avoidance, shapes vulnerability toward anxiety disorders. In support of this, BI children are at a significantly increased risk for anxiety disorders later in life (e.g. Chronis-Tuscano et al. 2009; Hudson and Dodd 2012) and this risk is exacerbated when their mothers support avoidance (Hudson et al. 2019). Early intervention programmes for BI children that lead to decreased long-term mental health problems focus on exposure exercises (decreasing avoidance) and parental support for exposure (Rapee et al. 2005). Similarly, in evidence-based treatments for child anxiety, avoidance behaviour is addressed via exposure exercises during which the child faces situations that evoke anxiety and fear (e.g. Kendall 1994). These exposure exercises are a central component of effective treatment (Bouchard et al. 2004; Kendall et al. 2005; Whiteside et al. 2020). Thus, exposure is a critical process for reducing risk for anxiety which needs to be targeted in effective treatment.

### Coping

Coping has been defined in a number of ways. Here we adopt the definition of Compas and colleagues that coping involves ‘efforts to regulate emotion, cognition, behaviour, physiology, and the environment in response to stressful events or circumstances’ (Compas et al. 2001, p. 89). Aligning with Eisenberg’s definition, coping is viewed as a subset of self-regulation (e.g. Eisenberg et al. 1997). Cognitive–behavioural theories for the treatment of child anxiety have coping at their core (Kendall 1994); Kendall describes that an effective treatment programme is one that leads a child to acquire a ‘coping template’ (Kendall 2011). This is defined as a cognitive structure for future events that incorporates adaptive skills and cognitions associated with adaptive functioning.

In support of the idea that maladaptive coping underpins childhood anxiety, clinically anxious children employ more maladaptive and less adaptive coping strategies in response to negative life events than non-anxious children (Legerstee et al. 2010). Specifically, studies in non-clinical community samples have shown that fearfulness is associated with greater use of maladaptive coping strategies characterised by rumination, self-blame and catastrophising and less use of adaptive coping strategies characterised by positive reappraisal (Garnefski et al. 2007). Clinically anxious children have also been found to ruminate more about the feelings associated with negative life events, and to focus more on catastrophic thoughts that emphasise the negative aspects of their experiences; they engage in less positive reappraisal of negative life events and are less able to refocus to plan what steps to take, and how to handle negative life events (Legerstee et al. 2010). Further, when faced with ambiguous scenarios, children higher in anxiety anticipate that they will experience more negative emotion than those with lower anxiety, indicative of reduced expectations of coping (Creswell and O’Connor 2011; Dodd et al. 2015; Waters et al. 2008). Evidence that coping plays at least a maintenance role, and possibly a causal role in anxiety comes from research demonstrating that improvements in coping mediate outcomes in psychological therapy, with children who show greater improvements in coping efficacy (the belief that they are able to actively cope with, rather than avoid anxiety-provoking situations) more likely to show improvements in anxiety across treatment (Kendall et al. 2016; Lau et al. 2010; Ollendick et al. 2017).

### Intolerance of Uncertainty

Recent theoretical and empirical work suggests that a fundamental fear of the unknown underpins anxiety (Carleton 2016a). Individual differences in fear of the unknown are captured by the construct of Intolerance of Uncertainty (IU). The most recent definition of IU is provided by Carleton (2016b) as “an individual’s dispositional incapacity to endure an aversive response triggered by the perceived absence of salient, key or sufficient information and sustained by the associated perception of uncertainty” (p. 31). Although early theory and evidence related to IU focused on worry and generalised anxiety disorder specifically, more recent work has shown that IU may in fact be a transdiagnostic factor that has relevance across anxiety disorders. For example, IU has been found to be significantly associated with social anxiety disorder (Boelen and Reijntjes 2009; Carleton et al. 2010), panic disorder (Carleton et al. 2014), post-traumatic stress disorder (Bardeen et al. 2013; Fetzner et al. 2013; Oglesby et al. 2016) and separation anxiety (Boelen et al. 2014). Importantly, treatments for anxiety disorders that specifically focus on exposure to uncertainty and cognitions around uncertainty, targeting IU, have been found to be effective in reducing symptoms of anxiety in adults (Hebert and Dugas 2019; van der Heiden et al. 2012) and changes in IU have been found to predict treatment outcome (Bomyea et al. 2015; Boswell et al. 2013a, b). Although developmental research evaluating the role of IU as a causal risk factor for anxiety has yet to be conducted, a recent meta-analysis of research into IU in child and adolescent anxiety and worry included 31 studies and showed a robust association, supporting findings from the adult literature that IU is strongly related to anxiety and worry (Osmanagaoglu et al. 2018).

### Interpretation of Physiological Arousal

Within a cognitive–behavioural framework, panic and agoraphobia are thought to be underpinned by catastrophic misinterpretations of bodily sensations such as “When my heart beats rapidly, I believe I’m having a heart attack” (Clark 1986). These misinterpretations lead to elevated fear and anxiety. Anxiety sensitivity (AS) refers to individual differences in this type of dysfunctional belief, or the tendency to fear arousal-related bodily sensations due to the belief that they are harmful (McNally 2002). AS is sometimes referred to as ‘fear of fear’. When an individual is high in AS, they are more likely to experience high levels of anxiety because they fear their physiological reaction as well as the threatening stimulus or situation. AS can be measured via self-report questionnaires in adults and children (e.g. Silverman et al. 1991; Taylor et al. 2007) and is linked to risk for anxiety disorder. For example, AS predicts anxiety symptoms and panic attacks as well as anxiety diagnosis more broadly, over time (Calkins et al. 2009; Hovenkamp-hormelink et al. 2019; McLaughlin and Hatzenbuehler 2009; Schmidt et al. 2006) and a meta-analysis of studies in children and adolescents demonstrated that AS is associated with a range of anxiety disorders (Noel and Francis 2011). Given this, there is a focus on developing treatments that target anxiety sensitivity. These demonstrate that, in adults, anxiety sensitivity can be changed and that this can have benefits for a range of anxiety-related mental health problems (Olthuis et al. 2014; Smits et al. 2008). Less research has been conducted with children and adolescents but initial evidence is consistent with these adult findings (Sherman et al. 2019). One of the ways that anxiety sensitivity is targeted in interventions is via Interoceptive Exposure (IE). IE involves exposure to the physical sensations that are associated with anxiety and fear (e.g. heart palpitations, sweating, muscle tension), with the view to increasing tolerance of these sensations and, in turn, reducing distress and fear related to these sensations (Craske and Barlow 2007). In IE, these physical sensations can be induced in a number of ways including via exercise, spinning and tensing the body (Boswell et al. 2013a, b).

## Child Individual Difference Factors Hypothesised to Affect Adventurous Play

While not a central focus here, individual differences between children provide a context within which our model sits. Child individual difference factors such as child age and development, physical ability, sex and temperament likely affect the extent to which children engage in adventurous play and may also moderate associations between adventurous play and anxiety risk.

In our model, we focus on early to middle childhood. However, even within this age range, we would expect child age, and stage of development (which in typically developing children is closely, but not always perfectly correlated with child age) to impact on engagement in adventurous play. While adventurous play and risk-taking activities have been observed in very young preschool children (e.g. 1–3 years of age; Kleppe et al. 2017), and across early and middle childhood (e.g. 4–13 years of age; Coster and Gleave 2008; Sandseter 2007; Sandseter et al. 2020), there is some evidence that the prevalence of engagement in adventurous play activities increases with child age (Kleppe et al. 2017; Sandseter et al. 2020). This is likely to be because with increasing age most children develop the more advanced gross and fine motor, cognitive and social-emotional skills necessary to support healthy risk-taking during adventurous play activities such as climbing trees, riding a bike at speed and play fighting with peers. We might also expect the association between adventurous play and anxiety risk to be moderated by child age; it seems likely that there will be stages of development, for example, when children are pushing for more autonomy (Kuczynski et al. 2018), where children might particularly benefit from the opportunity to play in an adventurous way.

Although we have focused here on early to middle childhood, adolescents may well also benefit from opportunities to engage in adventurous play. While adolescents are often perceived as not ‘playing’, they spend time engaged in self-directed activity for enjoyment, without any clear purpose, which is in keeping with definitions of play. Given that risk-taking increases during adolescence (Gardner and Steinberg 2005), the current model, which focuses on supporting healthy risk-taking in children’s play, may not be a good fit. Instead, for adolescents, it may be important to focus on providing an environment that allows healthy risk-taking with a view to decreasing unhealthy risk-taking and its associated physical and mental health correlates. This remains an issue to be explored in future theoretical and empirical work.

Child sex likely influences engagement in adventurous play as well as adult responses to and encouragement of their adventurous play. Boys and girls use playgrounds differently, although their overall activity levels are comparable; boys are more likely to play sports games and active games such as chase, whereas girls are more likely to be sedentary (sitting, reading, playing card games) or walking/running (Reimers et al. 2018). When it comes to adventurous play, Little (2010) found that, although boys and girls engaged in equivalent amounts of risk-taking in their play (defined as high risk, positive play), girls engaged in almost four times the amount of exploratory risk appraisal than boys. This indicates sex differences in children’s approach to engaging in risk-taking during play, with girls taking more time to evaluate risk level before engaging in adventurous play. This is consistent with research showing that boys take more risks in experimental play-based tasks than girls (Morrongiello and Sedore 2005) as well as data showing that boys experience a higher rate of injuries than girls (Hillier and Morrongiello 1998), and these injuries are often more severe (Morrongiello et al. 2004). It is important though not to make generalisations on the basis of child sex and to keep in mind that sex differences are likely determined by cultural and social norms (Liu and Zuo 2019), which means they may change with time and depending on context. There is some evidence to indicate changing gender patterns in children’s participation in organised sports activities with increased numbers of girls now participating in sports previously considered to be boys sports (e.g. football, cricket, basketball, rugby (ChildWise 2017). However, as yet, secular changes in boys and girls risk-taking during play have not been explored.

Parent behaviour also appears to differ depending on child sex, with mothers of daughters more likely to judge playground behaviour as risky and to intervene earlier and more frequently than mothers of boys (Morrongiello and Dawber 2000). It is well established that girls are at increased risk for anxiety during adolescence and adulthood (Collishaw et al. 2010; Remes et al. 2016). Therefore, given that they are also more likely to be actively discouraged from playing in an adventurous way, it may be particularly important to encourage adventurous play in girls. Given that boys may, in general, be more impulsive in their risk-taking during play and are more likely to injure themselves (Rosen and Peterson 1990), for them encouragement of more careful risk assessment may be valuable.

Individual differences in sensation seeking and behavioural inhibition also represent child-level factors that may influence engagement in adventurous play. Sensation seeking refers to an individual’s propensity to ‘seek varied, novel, complex and intense sensations and experiences, and the willingness to take physical…risks for the sake of such experiences’ (Zuckerman 1994). Although the construct was initially investigated in adults, it has subsequently been demonstrated to apply to children (e.g. Kafry 1982; Morrongiello et al. 2010). Sensation seeking is higher in male children then female children and is not associated with socio-economic status (Haas et al. 2019). Young children who are high in sensation seeking take more risks and are more likely to suffer minor and moderate injuries than those who are lower in sensation seeking (Morrongiello et al. 2010). Children who are high sensation seekers may create for themselves ample opportunities to play in an adventurous way and to learn about uncertainty, coping and arousal.

In contrast, behavioural inhibition (BI) reflects a heritable temperamental trait, that emerges early in life and is characterised by a tendency to respond to novel, unfamiliar persons, places and objects with withdrawal, wariness and avoidant behaviours (Kagan et al. 1988; Robinson et al. 1992). Compared to uninhibited children, inhibited children are fearful, shy and cautious and show increased signs of physiological arousal at rest (Fox et al. 2005). They are also at heightened risk for developing an anxiety disorder (e.g. Clauss and Blackford 2012; Sandstrom et al. 2020). Individuals who score high on behavioural inhibition may have less propensity to seek out adventurous play experiences. Given this, and the fact that these children are already at heightened risk for anxiety, the opportunity and support to play in an adventurous way may be particularly important as a means of lowering risk for anxiety. The same may be true for children who are at risk for anxiety because they have an anxious parent (e.g. Hudson et al. 2019).

## Environmental Factors Hypothesised to Affect Adventurous Play

A range of social and environmental factors affect children’s play broadly. These have been reviewed elsewhere (e.g. Whitebread et al. 2012) and are not a central focus here, but they include parent–child attachment, poverty, stress and nutrition (Lester and Russell 2010; Panksepp 2001). Assuming a safe context where these needs are met, adventurous play is likely affected by the affordances of the play space as well as the social environment, including the nature of adult supervision and the rules and policies in place (Fig. 1). In Europe and internationally, there is a concern that safety is often prioritised over children’s developmental needs, and that ‘surplus safety’ restricts children’s opportunities for adventurous play (e.g. Brussoni et al. 2012; Gill 2007; Tremblay et al. 2015) by constraining play spaces and children’s freedom to take risks when they play.

### Physical Environment

Children’s physical environments provide opportunity for different types of play and children perceive these affordances as invitations to play in a certain way (Heft 1988). Thus, the affordances offered by children’s physical environment are a vital characteristic in determining the extent to which children will engage in adventurous play. Research indicates that children spend little time ‘playing out’ in their local neighbourhoods compared to previous generations (Loebach and Gilliard 2016) and only a small minority of children play in natural outdoor spaces such as woodland (EnglandMarketing 2009). This is likely due to a range of factors including an increase in traffic and reduced access to natural spaces but it means that children’s opportunities to play adventurously are increasingly limited. Even purposely designed play parks are often poorly matched to children’s developmental needs and desires and are designed only with safety in mind. For example, Herrington and Nichols (2007) found that the Canadian standards for children’s playspaces and equipment did not reflect children’s developmental and play needs, but rather the goals of risk reduction. Similarly, Australian research found that children liked to play in a way that allowed them to experience height and speed (e.g. climbing ropes, slides etc.) but that parks provided few opportunities for children to master these skills or to learn new ones. Again, it was concluded that play spaces had been designed with a focus on safety and not on children’s preference for adventurous play (Little and Eager 2010).

Of course, there is no desire to create play spaces that are unsafe, but it is appropriate to consider whether play spaces can be designed that allow children to engage in adventurous play while ensuring safety standards are met. Natural-design playgrounds and/or adventure playgrounds may offer this balance, affording opportunities for risk-taking and thrilling adventurous play (Brussoni et al. 2012). Several studies have shown that adventurous play is increased in natural-design playgrounds. For example, Lee and Christiansen (1999) compared a traditional playground comprising swings, slides, see-saws, etc., with a natural-design playground containing wild, natural areas where wood and ropes were used. The natural design playground afforded the most challenging play, and on the traditional playground, children spent more time wandering and standing still. Children were also most enthusiastic about the natural design playground.

### Social Environment

In addition to the affordances offered by the play space, other people inspire or constrain action in the context of play (Gibson 1979). Kyttä (2004) uses the phrase ‘independent mobility licence’ to describe the extent to which adults allow children to move around freely and engage in the opportunities their environment offers. For children, this ‘licence’ is typically determined by adults in a caregiving capacity (parents or non-parent adults such as teachers and lunchtime supervisors). These adults may restrict children’s adventurous play via policy, rules and procedures and through their supervision of play. For example, in the UK, a number of playground games have been banned by schools because of health and safety concerns (Association for Teachers and Lecturers 2011). Similarly, in certain regions of Belgium, snowball fights have been banned (Sewell 2013). Research demonstrates that children’s opportunities to engage in the adventurous play opportunities afforded by their playground are affected by the mobility licence provided to children by supervising staff (Sandseter 2009).

Outside of school, parents have a vital role to play not only in protecting their children but also in encouraging them to take appropriate risks that support positive development (Kelley et al. 1998). The majority of children are supervised wherever they play (England Marketing 2009) and parental concern about child safety is one of the most significant influences on children’s independent outdoor play (Aziz and Said 2012; Valentine and McKendrick 1997). This monitoring of risk-taking by parents has been captured in laboratory research on sex differences which showed that parents who monitor their child more have children who take fewer risks (Hagan and Kuebli 2007). Furthermore, parent attitudes towards children’s risk-taking are correlated with their child’s engagement in adventurous play such that parents who feel more positive about the benefits of risk-taking in play have children who play more adventurously (Little et al. 2011). Research has also shown that peers can have an effect on children’s risk-taking behaviour. For example, research has shown that children engage in greater risk-taking in the presence of another child (Miller and Byrnes 1997; Morongiello and Sedore 2005), and that peer social norms influence children’s risk-taking decisions (Green and Hart 1998; Morongiello et al. 2013). Taken together, therefore, it is clear that parents, other adult caregivers and peers are likely to have some influence on their child’s risk-taking in play. Nevertheless, other child factors and environmental factors are also likely to play a role.

## Changing the Physical and Social Environment

Importantly, there is evidence that the above physical and social environmental factors can be changed, at least to some extent. For example, Bundy and colleagues changed children’s physical play environment at school by introducing materials with no fixed purpose (e.g. old car tyres, boxes, also known as loose parts) to a school playground for 11 weeks (Bundy et al. 2017). They found preliminary evidence that children’s play changed as a result, and that children spent more time engaged in physical activity. Alongside this, qualitative research shows that a risk-reframing intervention can help parents and educators to gain more realistic perceptions of risk and increase awareness of the benefits of age-appropriate risk-taking during play (Nieheus et al. 2013). Furthermore, there is qualitative evidence that programmes which aim to change the physical environment, adult attitudes to risk-taking and policies around play lead to changes in attitude and school culture around play, in particular in relation to risk-taking in play and adult control of play (Lester et al. 2011). To date, there is a lack of quantitative evaluations, using randomised control designs, of these programmes. Nevertheless, taken together, these studies provide an initial indication that it should be possible to increase children’s opportunities for adventurous play by making changes to their physical environment and by changing adult attitudes and behaviours related to child risk-taking.

## A Conceptual Model of How Adventurous Play might Affect Children’s Risk for Anxiety

Our conceptual model is shown in Fig. 1. It builds on theoretical accounts of the function of adventurous play and developmental psychopathological models of anxiety. Our model acknowledges that children’s engagement in adventurous play and the impact of adventurous play on children’s risk for anxiety will likely be affected by child individual difference factors, represented by the grey shaded area. It includes pathways through which adventurous play might affect children’s risk for anxiety as well as environmental and social factors that might affect each other as well as children’s engagement in adventurous play. Each of these paths is outlined in turn below. A direct, bidirectional path from adventurous play to anxiety symptoms is also included to acknowledge that the mediating risk mechanisms shown are unlikely to be exhaustive and that children with lower anxiety may be likely to engage in more adventurous play. There is also a path from decreased anxiety symptoms back to social environment to acknowledge that children’s anxiety is likely to affect the behaviour and attitudes of those around them. As outlined above, the model focuses on risk mechanisms that are linked to anxiety risk, are well-grounded in psychological theory and that can plausibly be affected by adventurous play.

We hypothesise that ample opportunities to engage in adventurous play during early and middle childhood will decrease children’s risk for problematic anxiety by providing children with exposure to fear-provoking situations during play which, in turn, provides opportunities for children to learn about coping, uncertainty and physiological arousal. Relative to play that is not adventurous, adventurous play exposes children to higher levels of uncertainty and risk which, in turn, exposes children to greater physiological arousal and fear. Successful handling of this uncertainty, arousal and fear will, we hypothesise, lead to an adaptive coping template, reduced intolerance of uncertainty and realistic interpretations of physiological arousal in the future (Kendall et al. 2016). Overcoming avoidance of fear-provoking situations through playful exposure is key to unlocking these benefits, and this is reflected in our model.

We hypothesise that adventurous play facilitates approach behaviour (decreases avoidance, increases exposure) because the positive, thrilling and playful emotion associated with this type of child-led play facilitates and motivates exposure to feared stimuli. This is supported by qualitative research showing that children are motivated to engage in play where they can experience the ambiguity of simultaneous exhilaration, joy and fear, or what they label “scary-funny” feelings. This motivated feeling comes from the balance between fear and fun. If the level of risk or fear is too high, the play will no longer be motivating and enjoyable. What constitutes adventurous play will therefore vary between children according to individual differences in propensity to take risks and experience fear; adventurous play happens when a child is playing at the edge of their comfort zone and experiencing these simultaneous feelings of excitement, thrill and fear.

### Supporting (Indirect) Evidence

Scientific research into the psychological benefits of adventurous or risky play in children is scarce. A systematic review conducted in 2015 identified 21 papers that examined associations between risky play and child health related outcomes. This review found significant, but mostly small, associations between risky play and increased physical activity as well as social competence but found no papers that evaluated mental health outcomes (Brussoni et al. 2015). Although the hypothesised association between adventurous play and anxiety in children has not yet been directly evaluated, there are three lines of research that provide evidence which is consistent with this theorised relationship: research on play in nature, research on the role of parents in child anxiety risk and research on sensation seeking. Each will be discussed briefly in turn.

### Outdoor Play in Nature

Outdoor, natural space lends itself to adventurous play and adventurous play is most likely to occur outdoors. This is because the diverse, dynamic and flexible features found in natural spaces afford many opportunities for adventurous play encouraging risk-taking, exploration, independence and autonomy (Bixler et al. 2002; Kellert 2002). As a consequence, there is overlap between adventurous play and both outdoor and nature play. These terms are not synonymous though; there are likely benefits to being outdoors and in nature that are independent of benefits that come from adventurous play in those spaces. Similarly, children can be outdoors but not playing in an adventurous way. And, with some creativity, adventurous play can happen indoors. Nevertheless, given this overlap, research on the mental health benefits of time outdoors and in nature is relevant. Broadly speaking, exposure to nature and green spaces is beneficial for children’s mental, physical and social health and well-being (e.g. Chawla 2015; Gill 2014; Mygind et al. 2019; Tillman et al. 2018). More specifically, when outdoor spaces in childcare settings and schools are ‘greened’ (modified to improve biodiversity and increase affordances and access to natural elements), this improves mental health and well-being, reduces stress, and promotes social and emotional skills (Bell and Dyment 2008; Brussoni et al. 2017; Chawla et al. 2014; Dankiw et al. 2020; van Dijk-Wesselius et al. 2018). It is theorised that these benefits come from the restoration of attention (see Kaplan and Kaplan 1989; Kaplan 1995) or stress reduction (see Ulrich et al. 1991) but it is also possible that some of this benefit comes from an increase in children’s adventurous play in these ‘greened’ spaces.

### The Role of Parents in Child Anxiety risk

When parents have higher levels of anxiety, their child is at increased risk for having an anxiety disorder (Turner et al. 1987). This association is particularly strong for maternal anxiety (Cooper et al. 2006). Overall, it also appears that when mothers are overprotective and attempt to protect their child from potential harm by controlling their behaviour, this increases children’s risk for anxiety disorders (Hudson et al. 2019; McLeod et al. 2007), although findings are somewhat mixed (van der Bruggen et al. 2008). In contrast, challenging parenting encompasses physical and verbal behaviours that encourage the child to stretch themselves physically and mentally through physical play and exposure to risk. It is theorised that fathers are particularly well-placed to engage in challenging parenting and that when they do they lower their child’s anxiety risk (Bogels and Phares 2008). There is some support for the hypothesis that challenging parenting decreases children’s anxiety risk, but findings regarding parent gender are less clear. For example, Lazarus and colleagues (Lazarus et al. 2016) found that both maternal and paternal challenging parenting were associated with lower anxiety symptoms but only maternal challenging parenting was associated with decreased risk for anxiety diagnosis.

This literature on parent anxiety and parenting styles is relevant to our conceptual model because, as discussed, a key environmental factor affecting children’s adventurous play is adult supervision, and the licence adults give children to take risks when they play. The extent to which parents encourage their children to engage in adventurous play and to take appropriate risks is very likely to be affected by the parents’ own anxiety and their parenting style. We would expect that parents who are highly anxious and/or more inclined toward overprotective, overcontrolling parenting may be more likely to restrict their children’s adventurous play via rules or more intense supervision and monitoring of play. In contrast, we would expect parents who display greater challenging parenting behaviours to actively encourage adventurous play and facilitate greater risk-taking via less intense supervision. Empirical research is lacking in this area although one study reported that parents who engaged in more overprotective parenting were less encouraging of their child engaging in adventurous play and perceived fewer benefits of adventurous play (Cevher-Kalburan and Ivrendi 2016).

### Research on Sensation Seeking

As outlined above, individuals differ in the extent to which they seek out risky and thrilling experiences, with individuals who are high on sensation seeking having a propensity to seek out more risky, exciting, thrilling experiences. Within our conceptual model, and as we have acknowledged above, individual differences in sensation seeking would represent a child-level factor that influences engagement in adventurous play. If the conceptual model is correct then these children should have ample opportunities to learn about uncertainty, coping and arousal and, as a result, have lower levels of anxiety. Evidence supports this link; children who are higher in sensation seeking have fewer internalising symptoms (Haas et al. 2019), which includes anxiety and depression. It seems likely that children who are high sensation seekers will not require the same level of environmental and social support to engage in adventurous play as children who are low sensation seekers. In fact, in extreme cases, it could be argued that risk-taking needs to be restricted in order to keep them safe. An alternative perspective would be that children who are high sensation seekers require an environment that matches their needs, rather than an environment that does not facilitate adventurous play and where it may be actively restricted by adults. Where this mismatch exists, we might anticipate behaviour problems as a result, which could offer some explanation for the correlation between high sensation seeking and conduct problems in children.

## Research Agenda

In the following paragraphs, we outline an agenda for future research that will allow our conceptual model to be evaluated. The first important step is to focus on how to measure children’s adventurous play reliably. The most direct means of measuring adventurous play is through the observation and coding of children’s play behaviours. Direct observation coding schemes have been developed for preschool children (e.g. Little 2010; Sandseter 2007), but these schemes may need adjusting to capture the adventurous play of older children, or to be appropriate for observing adventurous play in different environments. Given the subjectivity of what constitutes adventurous in different children, it may be necessary to incorporate some child self-report into observational measures of adventurous play. In addition to observational measures, which will provide in depth data but are time-consuming and costly, this area of research would benefit from a questionnaire measure that captures children’s opportunities for and engagement in adventurous play. Developing measures that capture the quantity (e.g. frequency, duration) and quality (e.g. location, degree of risk-taking) of children’s adventurous play are therefore an initial research priority.

Next, correlational studies incorporating measures of engagement in adventurous play, alongside measures of anxiety risk mechanisms, and anxiety symptoms can provide an initial ‘proof of principle’ test of our hypothesis. Such studies are relatively cost- and time-efficient, and if conducted with sufficiently large numbers of participants would allow some moderators to be examined. For example, it should be possible to explore whether the association between engagement in adventurous play and children’s anxiety differs for girls and boys. The results of these studies should be treated with some caution because it is likely that children who are more anxious will engage in less adventurous play (see Fig. 1) so a correlation could indicate this direction of effect.

Determining whether there is a causal relationship between adventurous play and decreased risk for child anxiety will require longitudinal and experimental research designs. Longitudinal designs can track adventurous play engagement, anxiety risk mechanisms, and anxiety symptoms across multiple time points and test whether the relationship between engagement in adventurous play and decreased anxiety risk occurs via the mediating risk mechanisms outlined in our model. Experimental designs where children are provided with opportunity for adventurous play with specific cognitive and behavioural factors evaluated before and after could provide an efficient and robust way to examine the causal hypotheses from the model. There are some limitations to both longitudinal and experimental designs though. For longitudinal research, the main limitation is that it will not be possible to rule out entirely that a confounding factor might affect both play and anxiety over time, leading to an association that is not causal. For experimental designs, the main limitation is that effects would only be found if adventurous play has an effect after only a short period. Furthermore, only state-like variables that change quickly over time could be evaluated for change from pre to post.

The most rigorous way to address questions of causality and to test the fundamental hypotheses in our model would be to conduct a randomised control trial in which children’s adventurous play is increased in an intervention group, and the effect on anxiety risk mechanisms and anxiety symptoms is compared with a control group. This will require the development of interventions that can successfully increase adventurous play, particularly in children who have the lowest levels of engagement in this type of play. Our model points to changes to the physical play space, and changes to adult attitudes and behaviours related to child risk-taking as key environmental factors that are likely to lead to increases in children’s adventurous play. There is evidence that these environmental factors can be changed effectively via modifications to school playgrounds, and via risk-reframing workshops, and that doing so leads to changes in children’s play, physical activity levels and well-being (e.g. Brussoni et al. 2017; Bundy et al. 2017; Lee and Christiansen 1999; Van Dijk-Wesselius et al. 2018) as well as to changes in parents and educators attitudes toward risk-taking in play (Niehues et al. 2013). However, as yet no randomised controlled studies exist that have manipulated adventurous play and examined the impact on child anxiety. If our conceptual model is correct, then we would expect that children who have increased opportunities for adventurous play will be at decreased risk of elevated anxiety symptoms because they are provided with exposure to and opportunities to learn about coping, uncertainty and physiological arousal. Not only would this research address important questions of causality but it would also have implications for prevention. Preventative interventions that increase adventurous play do not need to be highly resource-intensive, and even a small effect size of adventurous play on children’s anxiety may offer an opportunity to make a significant change in children’s risk for anxiety at a population level (Crutzen 2010).

Research that focuses on preventative interventions should ideally examine the impact of any intervention on anxiety risk within children who are at risk for anxiety disorders, for example those with a family history or anxiety or who have a behaviourally inhibited temperament. Because these children are at elevated risk for problematic anxiety, they have the most to gain from preventative interventions. With this in mind, it is important to evaluate whether adventurous play interventions are able to effectively change play and anxiety risk for these children specifically.

As we have noted, there is overlap between adventurous play and outdoor and nature play. There is also overlap with the constructs of free play, independent mobility, and physical activity. However, these constructs are not synonymous. We propose that there are likely benefits of adventurous play for children’s anxiety risk that operate via the mediating cognitive–behavioural pathways outlined in our model. From a theoretical perspective, it will therefore be important for future research to tease apart the effects of adventurous play from any effects due to time spent playing outdoors and in nature (e.g. Dankiw et al. 2020), of unstructured child-led play (e.g. Weikart 1998), of greater independent mobility (e.g. Marzi and Reimers 2018) and of higher levels of physical activity (e.g. Rodriguez-Ayllon et al. 2019; Zhu et al. 2019).

There are also important questions around moderators of any association between adventurous play and anxiety (e.g. child gender, child temperament, child age) as well as the hypothesised partial mediation pathways shown in Fig. 1. Both moderators and mediators could be examined in the context of large-scale survey studies and/or RCTs. It will be of particular interest to examine the impact of adventurous play on anxiety for children who are at increased risk for anxiety disorders, for example, children who have a family history of anxiety disorders or who have a behaviourally inhibited temperament. Initial research though should focus on the main hypothesis that engaging in more adventurous play will decrease children’s risk for elevated anxiety.

## Concluding Remarks

Until now the fields of child anxiety and adventurous/risky play have proceeded in isolation from each other despite good theoretical reasons to believe that they have relevance to one another. For the first time, in this conceptual article, we bring together these two research areas. We draw upon the literature regarding cognitive and behavioural factors that underpin child anxiety to propose a novel theory of how children’s risk for anxiety might be reduced by providing children with ample opportunity to play in an adventurous way. We set out an agenda for future research, identifying research priorities and designs that address the core hypotheses derived from our conceptual model. Through our model, and the research we hope it stimulates, there is significant potential to broaden our understanding of the benefits of adventurous play, moving beyond the dominant focus on physical health to also consider mental health outcomes, and to extend our understanding of the prevention of childhood anxiety.
